# The South African Pollen Monitoring Network: Insights from 2 years of national aerospora sampling (2019–2021)

**DOI:** 10.1002/clt2.12304

**Published:** 2023-11-01

**Authors:** Nanike Esterhuizen, Dilys M. Berman, Frank H. Neumann, Linus Ajikah, Lynne J. Quick, Erin Hilmer, Andri Van Aardt, Juanette John, Rebecca Garland, Trevor Hill, Jemma Finch, Werner Hoek, Marion Bamford, Riaz Y. Seedat, Ahmed I. Manjra, Jonny Peter

**Affiliations:** ^1^ Division of Allergology and Clinical Immunology Department of Medicine University of Cape Town Cape Town South Africa; ^2^ Evolutionary Studies Institute and School of Geosciences University of the Witwatersrand Johannesburg South Africa; ^3^ Unit for Environmental Sciences and Management Faculty of Natural and Agricultural Science North West University Potchefstroom South Africa; ^4^ African Centre for Coastal Paleoscience Nelson Mandela University Gqeberha South Africa; ^5^ Department of Plant Sciences Faculty of Natural and Agricultural Sciences University of the Free State Bloemfontein South Africa; ^6^ Smart Place CSIR Pretoria South Africa; ^7^ Laboratory of Atmospheric Science, Department of Geography University of Pretoria Pretoria South Africa; ^8^ Discipline of Geography University of KwaZulu‐Natal Pietermaritzburg South Africa; ^9^ Department of Otorhinolaryngology Gariep Mediclinic Kimberley South Africa; ^10^ Department of Otorhinolaryngology Faculty of Health Sciences University of the Free State Bloemfontein South Africa; ^11^ Hiway Medical Centre Westville Hospital Durban South Africa; ^12^ Allergy and Immunology Unit University of Cape Town Lung Institute Cape Town South Africa

**Keywords:** aeroallergens, aerobiology, aerobiomes, pollen, South Africa

## Abstract

**Background:**

Pollen monitoring has been discontinuously undertaken in South Africa, a country with high biodiversity, a seasonal rainfall gradient, and nine biomes from arid to subtropical. The South African Pollen Monitoring Network was set up in 2019 to conduct the first long‐term national aerospora monitoring across multiple biomes, providing weekly reports to allergy sufferers and healthcare providers.

**Methods:**

Daily airborne pollen concentrations were measured from August 2019 to August 2021 in seven cities across South Africa. Updated pollen calendars were created for the major pollen types (>3%), the average Annual Pollen Index over 12 months was calculated, and the results were compared to available historical data.

**Results:**

The main pollen types were from exotic vegetation. The most abundant taxa were Poaceae, Cupressaceae, Moraceae and *Buddleja*. The pollen season start, peak and end varied widely according to the biome and suite of pollen taxa. The main tree season started in the last week of August, peaked in September and ended in early December. Grass seasons followed rainfall patterns: September–January and January–April for summer and winter rainfall areas, respectively. Major urban centres, for example, Johannesburg and Pretoria in the same biome with similar rainfall, showed substantive differences in pollen taxa and abundance. Some major differences in pollen spectra were detected compared with historical data. However, we are cognisant that we are describing only 2 years of data that may be skewed by short‐term weather patterns.

**Conclusions:**

Differences in pollen spectra and concentrations were noted across biomes and between geographically close urban centres. Comparison with historical data suggests pollen spectra and seasons may be changing due to anthropogenic climate change and landscaping. These data stress the importance of regional and continuous pollen monitoring for informed care of pollinosis.

## 1 INTRODUCTION

The global incidence and severity of asthma and allergic diseases have increased,^1^, ^2^ decreasing the quality of life and causing, among others, a loss of productivity in the workplace^3^ and a decrease in the learning ability of school children.^4^ South Africa is witnessing a similar trend documented through the International Study of Asthma and Allergies in Childhood (ISAAC) Phase I to Phase II.^5^ Pollen is a major trigger for the seasonal symptoms of allergic rhinoconjunctivitis, and as pollen seasons can vary according to climate and location, there is a critical need to monitor pollen levels and the species prevalent in different regions. South Africa has nine distinct biomes containing numerous diverse vegetation units,^6^ each with its unique climate and suite of plant species. From a clinical perspective, allergists have long sought regional monitoring to better understand the pollen and fungal taxa driving respiratory allergies and to particularly understand regional differences. Unfortunately, to date, large and densely populated areas have either been completely unmonitored or last investigated in the late 1980s and 1990s.^7^, ^8^, ^9^ Furthermore, as anthropogenic climate change has been recognised as a major driver of changes in northern hemisphere aerospora, southern hemisphere data is urgently required given the major climatic, and landscape differences between hemispheres.^10^

Aerobiologist David Ordman pioneered aerobiology research in southern Africa from 1945 to 1972. His observations were prescient and subsequent pollen monitoring has confirmed many of his projections.^11^ Among other predictions, he suggested that allergenic trees in South Africa would include the popularly introduced ornamental trees of the cypress family (Cupressaceae)^12^ and he highlighted that patients might be sensitive to the dominant grass (Poaceae) types present in different regions of the country.^13^ Long‐term, continuous aerospora sampling has only been conducted in a single province and biome of South Africa: The Fynbos biome in the Mediterranean climate region of the Cape.^14^ Pollen monitoring was instituted by the Allergy Clinic at Cape Town's Red Cross Children's Hospital in the 1980s and in recent years has been continued at the University of Cape Town's Lung Institute.^15^, ^16^ Between 1987 and 1996, discontinuous pollen monitoring studies were conducted by aerobiologist Ann Cadman who sampled pollen in Cape Town (Fynbos biome), in the tropical coastal city of Durban in KwaZulu‐Natal (Indian Ocean Coastal Belt biome), and in the Savanna and Grassland biomes of the Gauteng province (Johannesburg and Pretoria).^7^, ^8^, ^9^ The latter research provided a strong platform for future pollen monitoring in the biomes of Gauteng.

**FIGURE 1:**
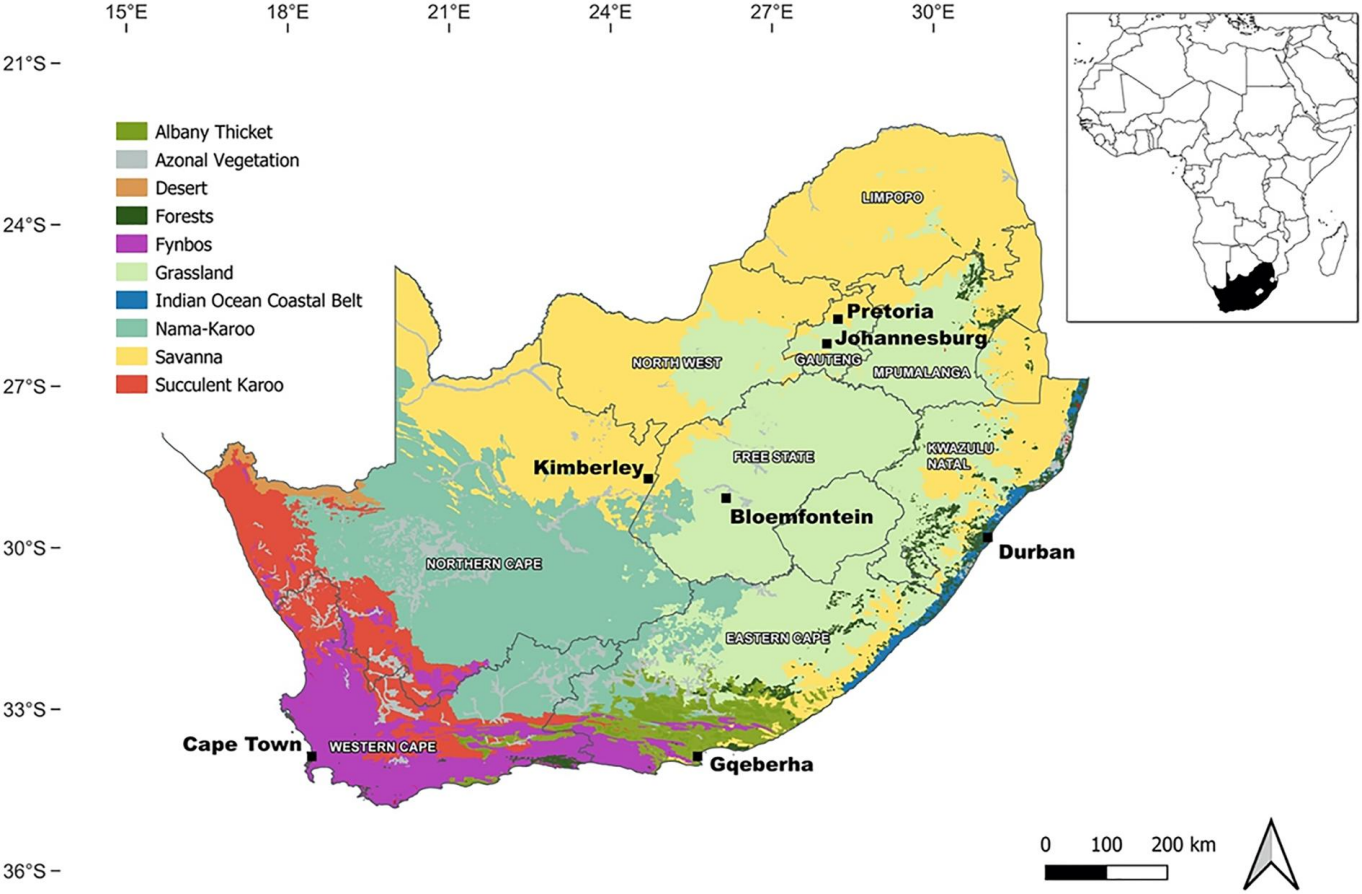
Map of the biomes of South Africa showing the seven cities (in black) where pollen monitoring was conducted from 2019 to 2021.^6^, ^18^

To extend pollen monitoring to more biomes and cities across the country, the South African Pollen Monitoring Network (SAPNET) was set up in August 2019 to conduct the first continuous aerospora sampling organised at a national level. Since its inception, SAPNET has monitored airborne pollen and fungal spores in seven of the major cities across South Africa, covering multiple biomes with a diversity of climates, topographies, and vegetation types (Ref. 16, 17; See Figure 1). An eighth site, Calvinia (a town in the Northern Cape province), was added to the network in 2021 and its results are not included here. Pollen and spore counts are conducted on a daily basis and the results are then shared online (www.pollencount.co.za) each week to provide an allergy risk guide for the public and healthcare providers.

This is the first analysis of the seasonal patterns and pollen spectra of the newly established pollen network, providing an overview of the findings of the first 2 years of comprehensive pollen sampling in South Africa. We highlight the major pollen types found at the different sampling locations, provide updated pollen calendars for each city and consider the future of aerobiome monitoring in South Africa. In light of climate change and predicted shifts in pollen seasons in the future,^19^ differences and similarities between the current findings and that of historical pollen monitoring at four of the sampling sites are discussed. Finally, the findings are contextualised within other southern hemisphere pollen monitoring networks to contribute to a broader understanding of global trends in the aerospora.

## 2 METHODS

### 2.1 Study sites

Daily airborne pollen concentrations were measured from August 2019 to August 2021 in seven cities across different biomes in South Africa: Cape Town (CPT, Fynbos biome), Johannesburg (JHB, temperate Grassland biome), Pretoria (PTA; Savanna biome), Bloemfontein (BFN; semi‐arid section of the Grassland biome), Kimberley (KMB; semi‐arid section of the Savanna biome), Durban (DBN; Indian Ocean Coastal Belt biome) and Gqeberha (formerly Port Elizabeth [PE]; Albany Thicket biome) (Figure 1). More information on each site can be found in Appendix Table A1.

South Africa's predominantly climatically derived biomes are diverse. The region is predominantly warm temperate, and coastal sites experience milder temperatures, while low winter temperatures occur in interior regions with snow on the high‐lying areas. The highest relative humidity is found in the humid‐subtropical regions (Durban site). The unique Fynbos biome in the Mediterranean climate zone of the Western Cape province (Cape Town site) is one of the world's six floral kingdoms with over 9000 plant species of which 70%–80% are endemic.^20^ Large areas of the country, particularly in the Northern Cape province (Kimberley site) are semi‐arid, featuring a variety of succulent plants, indigenous thorn trees and shrubs, and an increase in grasses after rains. The neighbouring province of the Free State (Bloemfontein site) lies in the Grassland biome and is characterised by an abundance of grass species, scarce tree cover and a semi‐arid climate, bordering on the semi‐arid region of the Nama Karoo. The subtropical climate of the Gauteng province's Highveld region (Johannesburg and Pretoria sites) is similarly characterised by grasses, while the Pretoria site, located as the southern fringe of the Savanna biome, features indigenous trees, for example, acacias. On the east coast of the country in the KwaZulu‐Natal province (Durban site), the humid‐subtropical climate supports a unique vegetation of the Maputaland‐Pondoland‐Albany global biodiversity hotspot. The oceanic climate of the Eastern Cape coastline (Gqeberha site) brings about its unique suite of plant species, as it is the confluence point of five of the nine biomes of South Africa. Afrotemperate forest cover is confined to small patches in the Western Cape province along the Great Escarpment and the eastern margins of South Africa, including the Tsitsikamma and Drakensberg forests. With regard to urban forests, Johannesburg is regarded as one of the most extensive man‐made urban forests globally.^21^, ^22^

Even though exotic trees in cities are a global phenomenon, the number of alien trees in South Africa is high due to optimal climatic conditions (both temperate and [sub] tropical trees thrive here) and the introduction of alien tree species is linked to the country's colonial past for example, oak, pine, poplar and plane trees. All the cities and their surroundings are inundated with alien invasive vegetation from other southern hemisphere countries (notably *Eucalyptus* and *Acacia* trees from Australia) and northern hemisphere locations (North America, Europe, Asia). More details on the common indigenous and exotic plant species at each site can be found in the Supplementary Material S1, together with a section on Method Limitations for consideration in future studies.

### 2.2 Sampling and pre‐processing

Standard aerospora monitoring devices, Hirst type 7‐day volumetric spore traps (manufactured by Burkard, UK), were used at all sites. Six of the seven locations had spore traps installed in 2019, while the Cape Town spore trap has been operational for the past 30 years. The height of the spore traps varied from 5 to 20 m above ground. Aerospora are continuously trapped/collected by the spore trap, using a constant air intake volume of 10 l/min and drum rotation of 2 mm/hr,^15^ to produce daily samples. Daily samples were then prepared and mounted on microscope slides for microscopic analysis using glycerol jelly, complying with the guidelines of the European Aerobiology Society Working Group on Quality Control.^23^ Three longitudinal traverses were read per slide, avoiding the margins. The number of individual aerospora counted along the three longitudinal traverses was totalled for each pollen or fungal taxa. This number was the raw score. The conversion of the raw score to the concentration of pollen grains or fungal spores per cubic metre was made as follows: The raw score was converted to the concentration per cubic metre of air per 24‐h period by applying the Correction Factor. The Correction Factor was calculated by dividing the total area of the strip (48 × 14 mm) by the area of the strip that was read, or the sum of the area of the three traverses that were read on each strip (48 mm × 14 mm × 0.45 mm × 3). This calculated figure was in turn divided by the volume of air sampled in a 24‐h period. The volume of air that was sampled or deposited on the strip in a 24‐h period was measured in cubic metres and equalled 14.4 m^3^ of air (10 L/min × 60 min × 24 h). This conversion is known as the Correction Factor (CF).^24^

Aerospora were identified based on published morphological studies and reference collections of fungal spores and pollen grains. Weekly pollen grain (pg) concentrations (data) for 2 years (104 weeks) of sampling were used to calculate the average Annual Pollen Index (API, pg/m^3^) and percentage pollen contribution to the different pollen categories (grass, trees, weeds) across South Africa. For the pollen calendars, the average weekly pollen index (pg/m^3^) was calculated using concentrations from the previous 14 days in a 52‐week calendar. If gaps in the dataset occurred in one of the years (due to technical issues with spore traps or Covid‐19 lockdown when traps were not operational, i.e., not true zeros), the pollen concentration from the alternative year's corresponding calendar week was taken as the average concentration. Out of 104 weeks, the following data were available at each site: Cape Town *n* = 98; Johannesburg *n* = 91; Pretoria *n* = 80; Bloemfontein *n* = 82; Kimberley *n* = 101; Durban *n* = 98; Gqeberha *n* = 93.

Pollen calendars displaying four coded levels of weekly pollen concentrations were created using Microsoft Excel. The levels were adapted from Potter and Cadman^25^ as follows: 0 = 0–3; 1 = 3–10; 2 = 10–30; 3 = 30–100; 4 = >100 pg/m^3^. Only the pollen types contributing more than 3% to the API of each monitoring site were included in this paper. The 3% cut‐off was chosen as pollen types that contributed <3% of the API fall below the significant threshold for allergy, that is, these taxa did not constitute a risk as triggers for allergic disease. This selection of taxa for inclusion in pollen calendars has been applied by other networks that published seasonal pollen calendars.^26^ An exception was made for Kimberley, where two plants below the 3% threshold were included due to their allergenic significance. Pollen Calendars that show the general tree, weeds, and grass seasons across the whole of South Africa can be seen in Supplementary Figure S1.

The start, peak, and end of the grass and tree pollen seasons for each site were calculated for the full winter‐to‐winter calendar year from July 1, 2020 to 30 June 2021 (see Appendix Table A2). The start of the grass season was defined as the first date after 1 July 2020 when daily pollen reached 10 pg/m^3^, the peak of the season was the highest daily pollen measurement between 1 July 2020 and 30 June 2021, and the end of the grass season was the last day of the 2020/2021 calendar when concentrations of 10 or more pg/m^3^ were detected. The tree season was defined similarly, except that the start and end dates were determined using 15 pg/m^3^ as the threshold value. The Cape Town spore trap power supply was faulty for a period during 2020/2021, resulting in missing data during the grass season and the peak tree season. Pollen measurements from 2010 to 2018 were thus used to calculate the Cape Town grass season start, peak, and end dates, as well as the peak date for trees.

## 3 RESULTS

### 3.1 Average Annual Pollen Index (API)

**FIGURE 2:**
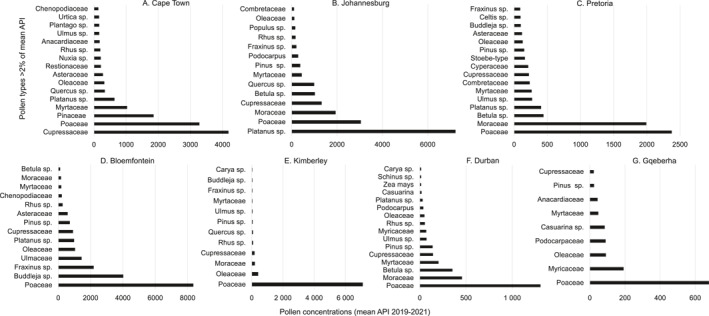
Ranked concentrations of the pollen types contributing >3% to the Annual Pollen Index (API) for (A) Cape Town, (B) Johannesburg, (C) Pretoria, (D) Bloemfontein, (E) Kimberley, (F) Durban, and (G) Gqeberha (PE, formerly Port Elizabeth) from August 2019 to August 2021.

**FIGURE 3:**
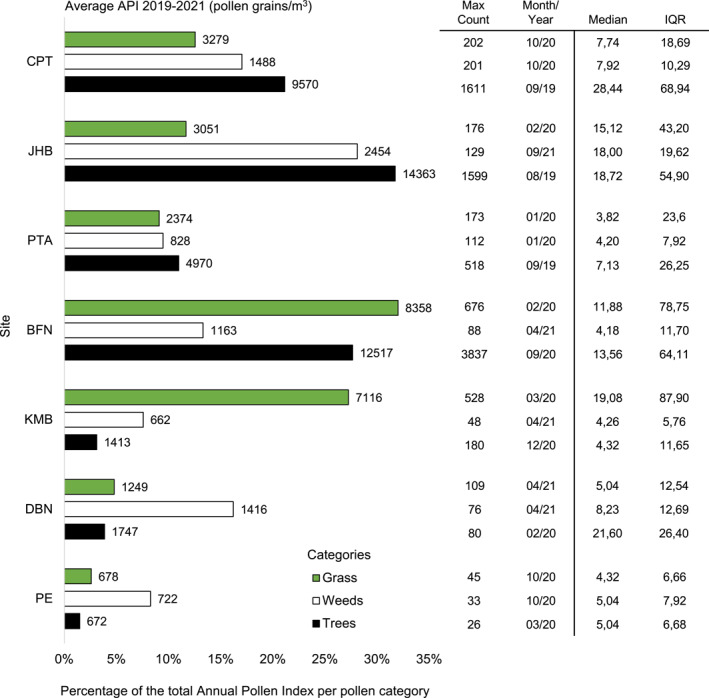
The average Annual Pollen Index (API, pg/m^3^) of the three pollen categories (grass, trees, and weeds) at each study site, shown as a percentage of the total amount of pollen recorded per category from 2019 to 2021. The total API of the pollen category per site is shown at the end of each bar. The maximum weekly concentrations are indicated in the table together with the date (month/year) when they occurred. The median weekly pollen concentration per pollen category and the interquartile range (IQR) are also shown.

Across all sites, Johannesburg had the highest average API for trees (14,363 pg/m^3^) and weeds (2454 pg/m^3^). The highest individual weekly concentration for trees was in Bloemfontein (3837 pg/m^3^ in September 2020) with the highest individual weekly concentration for weeds recorded in Cape Town (201 pg/m^3^ in October 2020). For grasses, the highest average API was in Bloemfontein (8353 pg/m^3^), which had the highest individual weekly concentration for this pollen category (676 pg/m^3^ in February 2020). Gqeberha (PE) was the city with the lowest tree, weed, and grass pollen concentrations (see Figure 2; Figure 3 for a complete site breakdown).

### 3.2 Pollen calendars

#### 3.2.1 Cape Town

**FIGURE 4:**
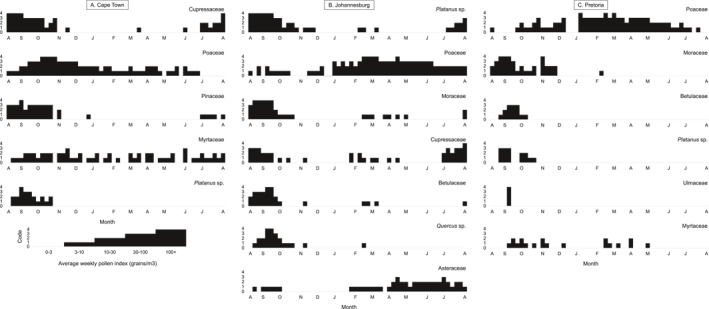
Pollen Calendars of the plant types contributing >3% to the Annual Pollen Index for (A) Cape Town, (B) Johannesburg, and (C) Pretoria. The average weekly pollen concentrations (2019–2021) are displayed as coded levels: 0 = 0–3; 1 = 3–10; 2 = 10–30; 3 = 30–100; 4 = >100 pollen grains/m^3^ (adapted from 23).

The pollen types contributing over 3% to the API in this city were cypress (Cupressaceae, 2092 pg/m^3^), grass (Poaceae, 1639 pg/m^3^), pine (*Pinus*, 928 pg/m^3^), myrtle (Myrtaceae, 515 pg/m^3^), and plane tree (*Platanus*, 319 pg/m^3^). The tree season started at the end of winter (July) when cypress and pine pollen increased to moderate and high levels throughout spring and early summer (August‐November). Myrtaceae pollen levels, including *Eucalyptus* species (gum tree), varied between low and moderate throughout the year, corresponding to flowering times of different myrtle species. The *Platanus* flowering season was shorter than the other trees, and plane tree pollen was recorded from late winter to summer (August to November) with a peak in spring (September). The main grass (Poaceae) season started in spring and peaked in early summer (October/November) (see Figure 4A). None of the weed pollen types recorded at this site contributed more than 3% to the average API. The most abundant taxon in this category was the daisy family (Asteraceae), which was also the most abundant weed pollen detected across South Africa (see Appendix Figure A1A). Other weeds included restios (Restionaceae), plantain (Plantaginaceae), nettles (Urticaceae) and goosefoot (Chenopodiaceae) (Appendix Figure A1B).

#### 3.2.2 Johannesburg

Plane tree (*Platanus*, 3613 pg/m^3^), grass (Poaceae, 1525 grains/m^3^), mulberry (*Morus*, 970 pg/m^3^), cypress (Cupressaceae, 662 pg/m^3^), birch (*Betula*, 512 pg/m^3^), oak (*Quercus*, 499 pg/m^3^), and the daisy family (Asteraceae, 342 pg/m^3^) were the main pollen types (>3%) detected at this site (see Figure 4B). The daisy family was the only weed type contributing over 3% to the API, but other minor weeds detected included the carnation family (Caryophyllaceae), goosefoot (Chenopodiaceae), slangbos (*Stoebe*‐type), and sedges (Cyperaceae) (Appendix Figure A1C). The grass season peaked in late summer (February/March), with elevated weekly concentrations recorded from early summer (January) until winter (June/July). The major tree pollen seasons stretched from early spring (August/September) to late spring (September/October), with the cypress and plane tree seasons starting earlier at the end of winter (July/August). Weed concentrations fluctuated throughout the year. Pollen from the daisy family was detected throughout the year; however, the highest concentrations were recorded in autumn (April/May) and winter (July).

#### 3.2.3 Pretoria

Grass (Poaceae, 1187 pg/m^3^), mulberry (*Morus*, 996 grains/m^3^), birch (*Betula*, 223 pg/m^3^), plane (*Platanus*, 202 pg/m^3^), elm family (Ulmaceae, 135 pg/m^3^), and the myrtle family (Myrtaceae, 132 pg/m^3^) were the plant types that made up over 3% of the API at this site (see Figure 4C). Short tree seasons with a peak in spring (September/October) were recorded for birch, plane, and elm. *Morus* pollen peaked in spring (September) and showed a second peak in early summer (November), whereas Myrtaceae pollen readings fluctuated throughout the year. The grass season peaked in early summer (January) and remained elevated until autumn (May). No weeds contributed more than 3% to the API, but the main pollen types detected were sedges (Cyperaceae), slangbos (*Stoebe*‐type), the daisy family (Asteraceae), and goosefoot (Chenopodiaceae) (Appendix Figure A1D).

#### 3.2.4 Bloemfontein

**FIGURE 5:**
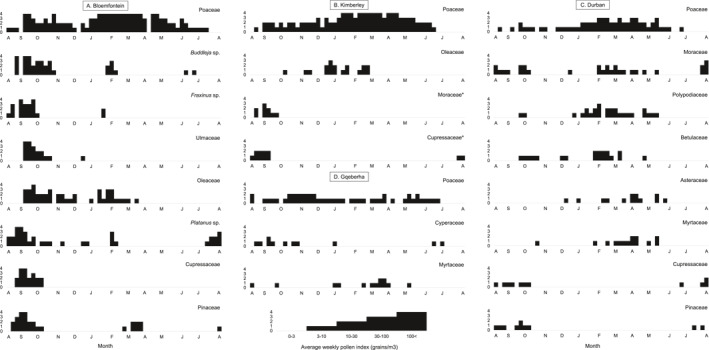
Pollen Calendars of the plant types contributing >3% to the Annual Pollen Index for (A) Bloemfontein, (B) Kimberley, (C) Durban, and (D) Gqeberha (formerly Port Elizabeth). The * indicates allergenic pollen contributions that missed the 3% cut‐off. The average weekly pollen concentrations (2019–2021) are displayed as coded levels: 0 = 0–3; 1 = 3–10; 2 = 10–30; 3 = 30–100; 4 = >100 pollen grains/m^3^ (adapted from 23).

High grass (Poaceae, 4179 pg/m^3^) concentrations dominated the pollen calendar at this site. Peaks were seen in spring (September/October) and throughout summer (January to March/April) with a decrease in grass pollen towards winter (May/June). Other pollen that made up more than 3% of the API, included false olive (*Buddleja*, 2008 grains/m^3^), ash (*Fraxinus*, 1079 pg/m^3^), elm family (Ulmaceae, 719 pg/m^3^), olive (*Olea*, 522 pg/m^3^), plane (*Platanus*, 492 pg/m^3^), cypress (*Cupressus*, 456 pg/m^3^), and pine (*Pinus*, 359 pg/m^3^) (see Figure 5A). Peaks for these pollen types varied; however, the main tree season started in early spring (August/September) and decreased towards summer (November/December). A second, smaller peak was recorded in later summer (February) for *Buddleja*, *Fraxinus*, *Olea*, and *Pinus*. Weed pollen did not contribute more than 3% of the API, but some notable weed species included the daisy family (Asteraceae), goosefoot (Chenopodiaceae), mugwort (*Artemisia*), and plantain (Plantaginaceae) (Appendix Figure A2A).

#### 3.2.5 Kimberley

Only two pollen types, grass (Poaceae, 3558 pg/m^3^) and the olive family (Oleaceae, 206 pg/m^3^), contributed more than 3% to the API (see Figure 5B). Two allergenic pollen types contributing just below this threshold were mulberry (Moraceae, 101 pg/m^3^) and cypress (*Cupressus*, 90 pg/m^3^). Grass was the most dominant pollen detected throughout the year, with the season peaking throughout summer (from January) and into autumn (April/May). Oleaceae showed three small peaks in early summer (November/December), midsummer (January/February), and late summer (February/March). The two allergenic tree pollen types included here, mulberry and cypress, both had short seasons between late winter (August) and early spring (September/October). No weed pollen contributed more than 3% to the API. The main weed species detected at this site were borages (Boraginaceae), common ferns (Polypodiaceae), goosefoot (Chenopodiaceae), the ice plant family (Aizoaceae), and daisies (Asteraceae) (Appendix Figure A2B).

#### 3.2.6 Durban

At this site, the pollen types that contributed more than 3% to the API, included grass (Poaceae, 656 pg/m^3^), mulberry (Moraceae, 228 pg/m^3^), common ferns (Polypodiaceae, 209 pg/m^3^), birch family (Betulaceae, 177 pg/m^3^), the daisy family (Asteraceae, 107 pg/m^3^), myrtles (Myrtaceae, 102 pg/m^3^), cypress (*Cupressus*, 72 pg/m^3^) and pine (*Pinus*, 70 pg/m^3^) (see Figure 5C). Grass concentrations increased in early summer (January) with small peaks occurring throughout late summer (February/March) until late autumn (May). Tree pollen was detected throughout the year. The peak *Morus* concentration was seen in late winter (August), with smaller peaks in spring (October) and late summer (February/March). *Betula* and Myrtaceae pollen was elevated between spring (September/October) and autumn (May), whereas *Cupressus* and *Pinus* pollen were elevated between late winter (July) and late spring (October). The two weeds included here both had increased concentrations between summer (December/January) and late autumn (May/June). Other notable weeds recorded at this site, albeit at levels lower than the 3% threshold, included ragweed (*Ambrosia*), sedges (Cyperaceae), nettles (Urticaceae), mugwort (*Artemisia*) and goosefoot (Chenopodiaceae) (Appendix Figure A2C).

#### 3.2.7 Gqeberha (PE)

Only three pollen types contributed more than 3% to the API at this site. Grass (Poaceae, 339 pg/m^3^) occurred at low to moderate levels throughout the year, with no clear seasonal detection. Similarly, sedges (Cyperaceae, 106 pg/m^3^) were present at low levels at different times of the year, mainly in winter (June/July) and again in spring and summer (August to November, and January) (see Figure 5D). Pollen from the myrtle family (Myrtaceae, 96 pg/m^3^) was also present in low to moderate levels throughout the year, with no clear seasonality. Besides sedges, no other significant weed concentrations were recorded. The other main weed pollen types included the daisy family (Asteraceae), the erica family (Ericaceae), the carnation family (Caryophyllaceae), slangbos (*Stoebe*‐type) and the protea family (Proteaceae) (Appendix Figure A2D).

## 4 DISCUSSION

Despite its high biodiversity, the main pollen types found during this 2‐year study in South Africa were from exotic vegetation. In an urban environment, wind‐pollinated plants (including many neophytes) are more common and within this landscape, specific climatic conditions such as wind speed often favour wind transport over biotic pollination.^27^ Specifically, indigenous trees were not well represented in the pollen spectrum as the pollinators of indigenous plant species are often insects, birds, or small mammals.^28^ Exotic northern hemisphere trees included allergenic trees such as *Platanus*, *Quercus*, *Morus*, *Fraxinus,* and *Betula*. Often these trees are not only grown as ornamentals in urban areas but, like *Morus*, can invade roadsides, riverbanks, and open spaces.^29^ Notable indigenous trees included *Olea europaea* subsp. *africana*,^30^ which was dominant in Bloemfontein and Kimberley, and *Buddleja*, which featured predominantly in Bloemfontein.

The tree pollen catch at all sites was low compared with some northern hemisphere cities. Daily *Betula* pollen loads increased to over 4000 in the Polish city of Kraków in 2016.^31^ In our study, maximum daily tree pollen peaks were less than 1500 for *Platanus* in Johannesburg and *Buddleja* in Bloemfontein. Despite the lower concentrations, symptoms of allergic disease are likely to be triggered by tree pollen during the August‐October spring season in Johannesburg, Bloemfontein, Cape Town, and Pretoria, and peaks for Oleaceae (*Olea* sp.) in Kimberley in the late summer months of December‐February are noted. Moreover, with anthropogenic climate change affecting pollen seasons and plant growth as temperatures and CO_2_ levels increase, shifts in the flowering times of trees, grasses and other allergenic plants are already noted, potentially favouring an increase in allergenic grasses and weeds.^10^, ^19^

Weed concentrations were generally low throughout the sampled cities; however, the major pollen types featured some exotic plants including plantains (Plantaginaceae) and nettles (Urticaceae) (see Appendix Figure A1A). Prominent species include invaders such as *Plantago lanceolata*, *P. major, Urtica dioica* and *U. urens* spreading in gardens and along roadsides.^29^ The main weed pollen detected was from the daisy family (Asteraceae), which includes many indigenous plant species such as Slangbos (*Stoebe*‐type). Other families frequently identified were the carnation family (Caryophyllaceae), the goosefoot family (Chenopodiaceae), sedges (Cyperaceae), the erica family (Ericaceae), and fern spores (Polypodiaceae). Notably, the allergenic weed *Ambrosia* has been detected at the Durban site, albeit in small numbers; however, its presence and any increase in the amount of pollen over time will be closely monitored.

The findings from this study highlighted differences in pollen spectra between sites in different regions of the country, and between geographically close sites, for example, Pretoria and Johannesburg or Kimberley and Bloemfontein. The latter two cities are 165 km apart and both cities have a semi‐arid climate. Kimberley in the Savanna biome had the lowest weed catch in the network (7.6%), with Bloemfontein in the Grassland biome having comparably higher weed concentrations (13.3%). Tree pollen concentrations in Kimberley (3.1%) were lower than in Bloemfontein (27.7%); however, their grass concentrations were comparable (15.7% and 18.5%) (see Appendix Figure A3).

The highest tree pollen concentrations were found in Johannesburg (31.7% of the total tree catch in South Africa), which has been extensively planted with exotic and indigenous trees, forming the largest man‐made urban forest in the southern hemisphere.^22^ The dominant pollen at this site was the allergenic tree *Platanus* (plane tree) (36.3% of the API; Table 1), which produced >100 pollen grains/m^3^ in August and September (pollen index 4) (Figure 4B). Interestingly, plane tree pollen was not recorded in the top pollen types found in Johannesburg in a study by Cadman in the 1980s.^7^ In their study, the most dominant pollen was grass (Poaceae, 50.82%), followed by pine (*Pinus*, 10.78%) (Table 1). When comparing findings from the present study to those of previously published pollen calendars for South Africa,^25^ we see that *Platanus* had an insignificant and short flowering window in August 1987–1988. This contrasts with the higher measurements and longer season (August to October) observed in the new calendar (see Figure 4B). *Platanus acerifolia* (the London plane tree) is, next to *Jacaranda mimosifolia* (Bignoniaceae), the most common tree lining avenues in Johannesburg.^32^ The Johannesburg grass season in the present study increases and fluctuates between pollen indexes 2 and 3 from January onwards, peaking in March (pollen index 4) and staying significant until mid‐winter. This indicates a longer and more continuous grass season than that found in the 1996 study by Potter and Cadman,^25^ where two fluctuating peaks were seen: one from October to January and another in May.

**TABLE 1:** Comparison between past and present annual pollen concentrations for the cities of Cape Town, Johannesburg, Pretoria, and Durban.

Cape Town						Johannesburg					
1993			2019–2021			1987–1988			2019–2021		
Pollen taxa	Average API	% Of total API		Average API	Pollen taxa	Pollen taxa	Average API	% Of total API		Average API	Pollen taxa
*Quercus*	606	22.6	28.87	4184.4	Cupressaceae	Poaceae	6454.5	50.82	36.33	7225.5	*Platanus*
Cupressaceae	505	18.83	22.62	3278.6	Poaceae	Pinaceae	1369.5	10.78	15.34	3050.5	Poaceae
Poaceae	457	17.04	12.81	1856.3	Pinaceae	*Populus*	670	5.28	9.76	1940.8	Moraceae
Pinaceae	341	12.71	7.1	1029.6	Myrtaceae	*Fraxinus*	654.5	5.19	6.66	1325.3	Cupressaceae
*Platanus*	144	5.37	4.4	638.4	*Platanus*	Eucalyptus	659	5.15	5.15	1023.9	Betulaceae
Oleaceae	88	3.28				Stoebe‐type	508	4	5.02	997.7	*Quercus*
						Asteraceae	414.5	3.26	3.44	683.5	Asteraceae

Pretoria						Durban					
1987–1988			2019–2021			1989–1991			2019–2021		
Pollen taxa	Average API	% Of total API		Average API	Pollen taxa	Pollen taxa	Average API	% Of total API		Average API	Pollen taxa
Poaceae	7604.5	63.86	28.31	2374.1	Poaceae	Poaceae	1447	34.6	28.9	1311.9	Poaceae
*Celtis*	1138.5	9.56	23.76	1992.2	Moraceae	*Cannabis*	836	20	10.05	456.4	Moraceae
*Fraxinus*	501.5	4.21	5.29	443.9	Betulaceae	Moraceae	418	10.3	9.2	417.6	Polypodiaceae
Ulmaceae	437.5	3.67	4.83	404.8	*Platanus*	*Celtis*	347	8.3	7.79	353.6	Betulaceae
Betulaceae	402	3.38	3.23	270.7	Ulmaceae	Asteraceae	178	42	4.7	213.4	Asteraceae
Myrtaceae	371	3.12	3.15	263.9	Myrtaceae	Cyperaceae	142	3.4	4.48	203.3	Myrtaceae
									3.17	143.8	Cupressaceae
									3.07	139.4	Pinaceae

*Note*: The pollen taxa contributing >3% to the total Annual Pollen Index (API) are indicated. Past data are taken from 7, 8, 9.

Johannesburg in the Grassland biome and Pretoria in the Savanna biome are sites located in close geographic proximity (60 km apart). The API for grass in Johannesburg was higher than in Pretoria (3051 vs. 2374 grains/m^3^); however, grass was the main pollen type detected in Pretoria. From the present data, Pretoria grasses peak from mid‐summer to May (pollen index 3 and 4) with a tail towards winter (Figure 4C). The Potter and Cadman^25^ calendars showed a similar long season with a fluctuating peak in November/December and increased concentrations until May/June. The second‐most dominant pollen type on the present Pretoria pollen calendar is a mulberry tree (*Morus*). In the past, a small peak in mulberry pollen was seen from July to August,^25^ compared to the current data, which shows a steady increase from August until September when a significant peak occurs (see 28). Another second, isolated peak of *Morus* was seen in November.

Cape Town has a Mediterranean climate, and this is reflected in its highest‐ranking pollen, *Cupressus* (28.87% of the API), with species such as *Cupressus sempervirens*, a wind‐pollinated and drought‐adapted ornamental from the eastern Mediterranean.^13^ This pollen type dominated the pollen spectrum in August and September (Figure 4A, pollen index 4)—even exceeding grass (Poaceae) concentrations. This pattern confirms the results of an earlier study at the same site (^33^, compare 13) but is different from a 1993 study by Cadman and Dames^9^ where cypress contributed 18,83% to the API and the most dominant pollen type was that of oak (*Quercus*, 22.6%) (see Table 1). In the calendars published by Potter and Cadman,^25^ oak peaked from July/August to September and cypress showed an insignificant peak from May to August only. In comparison, the present study shows cypress to be dominant and to have a long season that peaks between August and October.

In Cape Town, overall grass levels were lower in the Potter and Cadman^25^ study; however, the proportion of the grass index made up of the total API was comparable: 22.6% in the current study and 18.8% previously. However, archived grass proportions from 2010 to 2014 as a composite of the grass TPI at the same sampling site in Cape Town and a coastal site, Table View, was as high as 38%.^15^ Potter and Cadman found the peak grass flowering months to be October‐November and these were confirmed in our SAPNET study, with a steady increase from September towards this peak, whereafter the grasses decreased as summer progressed.

A lower API was seen from the coastal sites of Durban (subtropical summer rainfall) and Gqeberha (aseasonal rainfall) than from the inland cities (Figure 2, Appendix Figure A3). Low pollen concentrations in coastal cities have been shown by other pollen networks.^34^ Although Cape Town is a coastal site, it does not follow this general rule as it is situated in a winter rainfall region (Mediterranean climate). Grass flowering seasons differed according to the climate. Cape Town is a winter rainfall area where the grass season begins in late September and peaks in October, decreasing quickly in January. The inland sites of Johannesburg, Pretoria, Bloemfontein, and Kimberley are summer rainfall regions; thus, the grass season begins in January and declines in May‐June. In the coastal city of Gqeberha, the grass season was similar to that of Cape Town, where grasses increased from September to November (Figure 5D).

In subtropical Durban located in the Indian Ocean Coastal Belt biome,^6^ the daily average grass concentration seldom exceeded 30/m^3^; however, grasses did increase in January. Grass (Poaceae) was the top pollen type detected in Durban in both the past (34.6%; 8) and this present study (28.9%) (see Table 1 for further comparison). Peaks in our current grass pollen calendar are higher than those in the 1989–1991 study^8^, ^25^ and the season has become longer. Historically, grass pollen concentrations increased in summer to peak from January to March—the present results concur with these findings. However, the present grass season continues with some fluctuations until autumn, after which the levels decrease in June. Moraceae, the next dominant pollen type found in Durban, had a short season between August and September in the past. In the present study, mulberry similarly peaked at this time with multiple smaller peaks dispersed throughout the year (Figure 5C). However, our observations are based on 2 years of data collection only and we are cognisant that these could be abnormal conditions; our intention is not to mislead but merely to describe what we have collected in the initial 2 years.

The relationship between climate and pollen is evident when considering the differences observed in the grass seasons of sites across climate gradients. However, several changes in seasonality, plant dominance, and pollen levels over the past 30 years have been highlighted. Differences between historical and current data could be explained by natural and man‐made changes in the urban vegetation cover over time, for example, more ornamental trees planted in ever‐expanding suburbs and mature trees reaching flowering age, or environmental legislation around compulsory control and eradication of listed species. It should be noted that differences between historic and current sampling locations within the cities could be a contributing factor. Moreover, the effect of anthropogenic climate change and resulting shifts in plant flowering phaenology^35^ on past and present pollen seasons cannot be discounted. Therefore, considering predicted changes in climate and seasonal rains to come (e.g., Ref. 36) continued long‐term pollen monitoring with the addition of more sites will become increasingly important to predict changing allergy seasons in the future.

Anthropogenic climate change has an immediate effect on aerospora production worldwide, which will negatively affect human health.^37^ CO2 triggers plant production, causes seasonal shifts and might also alter the allergen content. More research is needed to understand aeroallergens in the context of the southern hemisphere; aerobiological studies from Australia and South America can now be compared with the current study from South Africa.

Darwin, situated in tropical northern Australia, shows a pollen peak in April and May with a high Poaceae, *Acacia* and Arecaceae pollen load at the beginning of the dry season, which ends in October.^38^ It is also mentioned that in Darwin, in contrast to more southerly temperate cities, which feature many exotics, indigenous Australian taxa are prevalent. Consequently, in temperate Australian cities like Perth, Sydney, Canberra, Melbourne and Hobart, next to Poaceae (peak pollen season spring‐summer) mostly Northern Hemisphere exotics like Cupressaceae, *Pinus*, *Olea*, *Betula* are the most abundant pollen taxa, but other exotics like *Ulmus*, *Quercus* as well as Northern Hemisphere weeds like *Plantago* are also fairly common outnumbering indigenous Australian taxa.^39^ For all of these taxa, the austral spring and summer (and to a lesser degree winter) are the peak pollen seasons in temperate/subtropical Australia not unlike the South African cities investigated in our study (compare 38).

Seemingly, at least for southern Africa and Australia as well as for temperate southern New Zealand, which shows similar pollen abundancies and trends throughout the seasons, it is obvious that exotic Northern Hemisphere trees, and to a lesser degree, weeds with often high allergenicities are much more prevalent than indigenous pollen taxa and reach pollen peaks mainly during austral spring and summer.^39^ Also in mostly warm temperate Argentina, the spring season was the most prevalent pollen season for grasses, trees and weeds.^40^ In several cities, grass pollen peaked in November‐December (late spring early summer),^41^ a few months earlier than for example, in Gauteng, where grass peaked from late summer until June (winter). In Mar del Plata (Argentina), Northern Hemisphere exotics, such as *Acer*, *Juglans*, *Betula*, *Salix*, *Fraxinus*, *Platanus*, and *Juglans*, were abundant during austral spring, whereas grasses started to increase in October‐November.^42^ The observed pattern is that Northern Hemisphere exotic trees are the most prolific pollen dispersers in spring in Southern Hemisphere warm temperate continental regions from South America to southern Africa and Australia.

## 5 CONCLUSIONS

After 2 years of monitoring (2019–2021) in seven cities, the main pollen types found in South Africa were from exotic vegetation—specifically wind‐pollinated northern hemisphere trees. Weed concentrations were generally low compared with those of trees and grasses, with the grass pollen calendar found to be influenced by seasonal rainfall. The main findings from this study show some similarities however major differences in pollen spectra detected in previous studies at the same sites, and some differences in pollen seasons were seen when comparing past data to this present study. These changes in seasonality over time and the differences observed in pollen spectra of geographically closely located sampling sites emphasise the need for continued pollen monitoring—not only across the existing sites but also at new locations, to provide effective healthcare to patients across South Africa. The general pattern of overwhelmingly Northern Hemisphere exotic trees in the atmosphere above southern Africa is in agreement with similar observations on other southern hemisphere continents, at least in the subtropical and temperate regions. This underlines that the spread of North American and Eurasian trees in city urban spaces should be better managed by South African government bodies to counter the increase in seasonal allergenic pollen.

## AUTHOR CONTRIBUTIONS

**Nanike Esterhuizen:** Conceptualization (equal); data curation (lead); formal analysis (lead); investigation (lead); project administration (lead); visualization (lead); writing – original draft (lead); writing – review & editing (equal). **Dilys M. Berman:** Conceptualization (lead); investigation (lead); project administration (lead); writing – original draft (equal); writing – review & editing (equal). **Frank H. Neumann:** Conceptualization (equal); investigation (equal); writing – original draft (equal); writing – review & editing (equal). **Linus Ajikah:** Investigation (equal); writing – original draft (equal); writing – review & editing (equal). **Lynne J. Quick:** Investigation (supporting); visualization (equal); writing – original draft (equal); writing – review & editing (equal). **Erin Hilmer:** Investigation (equal); project administration (equal); writing – review & editing (equal). **Andri Van Aardt:** Investigation (supporting); project administration (equal); writing – original draft (equal); writing – review & editing (equal). **Werner Hoek:** Investigation (supporting); project administration (equal); writing – original draft (equal); writing – review & editing (equal). **Juanette John:** Investigation (supporting); project administration (equal); writing – review & editing (equal). **Rebecca Garland:** Investigation (supporting); writing – original draft (equal); writing – review & editing (equal). **Trevor Hill:** Conceptualization (equal); writing – original draft (equal); writing – review & editing (equal). **Jemma Finch:** Writing – original draft (equal); writing – review & editing (equal). **Marion Bamford:** Conceptualization (equal); writing – review & editing (equal). **Riaz Y. Seedat:** Conceptualization (equal); writing – review & editing (equal). **Ahmed I. Manjra:** Conceptualization (equal); writing – review & editing (equal). **Jonny Peter:** Conceptualization (lead); funding acquisition (lead); supervision (lead); writing – original draft (equal); writing – review & editing (equal).

## CONFLICT OF INTEREST STATEMENT

The authors declare that this research was made possible by the following funders and industry affiliations: NE was funded by a UCT Lung Institute Postdoctoral Research Fellow award (2019–2022); RG and JJ were supported by a CSIR Parliamentary Grant for the duration of the project; The University of the Free State team received funding in 2020/2021 from the Interdisciplinary Funding provided by the UFS; This project was supported by Zeiss South Africa which sponsored light microscopes for aeropalynology at the University of Cape Town and the University of the Witwatersrand. The Real Pollen Count received industry sponsorship from Clicks, Dr. Reddy's, Thermo Fisher Scientific, Novartis, Glenmark, SA Natural Products, and Twinsaver.

## Supporting information

Supporting Information S1

Supporting Information S2

## Data Availability

The data that support the findings of this study are available from the corresponding author upon reasonable request.
