# Supplementary material for: The South African Pollen Monitoring Network: Insights from 2 years of national aerospora sampling (2019–2021)
Source: Clin Transl Allergy. 2023 Nov 1;13(11):e12304. doi: 10.1002/clt2.12304 (PMC10620116; doi:10.1002/clt2.12304)
Supplement: Supplementary file 1 — Supporting Information S1 [file CLT2-13-e12304-s001.docx]

### **Appendices**

**Appendix Table 1:** The seven cities in South Africa where pollen monitoring was conducted from 2019 to 2021.

| **City** | **Province** | **Biomes** | **Köppen Climate** | **Average Rainfall** | **Average  Temperatures** | **Elevation** | **Population** | **GPS Coordinates** |
| --- | --- | --- | --- | --- | --- | --- | --- | --- |
| Cape Town | Western Cape | Fynbos | Mediterranean | Winter  515 mm | Min 11.4 °C  Max 22.0 °C | 0 - 1,590.4 m | 4.7 million | 33°55′31″S  18°25′26″E |
| Johannesburg | Gauteng | Grassland | Subtropical  highland | Summer  713 mm | Min 10.1 °C  Max 21.9 °C | 1753 m | 8 million | 26°12′16″S  28°2′44″E |
| Pretoria | Gauteng | Savanna | Subtropical | Summer  673 mm | Min 12.3 °C  Max 24.7 °C | 1403 m | 2.9 million | 25°44′46″S  28°11′17″E |
| Bloemfontein | Free State | Grassland | Semi-arid | Summer  559 mm | Min 7.5 °C  Max 24.4 °C | 1395 m | 0.7 million | 29°07′S  26°13′E |
| Kimberley | Northern Cape | Savanna | Semi-arid | Summer  414 mm | Min 10.9 °C  Max 26.1 °C | 1184 m | 0.2 million | 28°44′18″S  24°45′50″E |
| Durban | KwaZulu-Natal | Indian Ocean  Coastal Belt | Humid-  subtropical | Summer  1019 mm | Min 16.5 °C  Max 25.2 °C | 8 m | 3.4 million | 29°53′S  31°03′E |
| Gqeberha  (PE) | Eastern Cape | Albany  Thicket | Oceanic | Year-round  624 mm | Min 13.5 °C  Max 22.3 °C | 60 m | 1.1 million | 33°57′29″S  25°36′00″E |

**Appendix Table 2:** Grass and tree season start/peak/end dates in South Africa for the year 1 July 2020-30 June 2021. The start of the grass season was defined as the first date after 1 July 2020 when daily pollen reached 10pg/m^3^, the peak of the season was the highest daily pollen measurement between 1 July 2020 and 30 June 2021, and the end of the grass season was the last day of the 2020/2021 calendar when concentrations of 10 or more pg/m^3^ were detected. The tree season was defined similarly, except that the start and end dates were determined using 15 pg/m^3^ as the threshold value.

|  | **Sampling site** | **Start date** | **pg/m^3^** | **Peak date** | **pg/m^3^** | **End date** | **g/m^3^** |
| --- | --- | --- | --- | --- | --- | --- | --- |
| Grass season | Cape Town* | 14 September | 12 | 25 October | 42 | 16 January | 12 |
|  | Johannesburg | 30 August 2020 | 23 | 17 February 2021 | 41 | 2 May 2021 | 12 |
|  | Pretoria | 27 October 2020 | 17 | 29 October 2020 | 32 | 20 April 2021 | 13 |
|  | Durban | 8 March 2021 | 11 | 6 April 2021 | 23 | 16 May 2021 | 10 |
|  | Bloemfontein | 7 September 2020 | 78 | 30 October 2021 | 82 | 16 March 2021 | 25 |
|  | Kimberley | 3 October 2020 | 20 | 1 April 2021 | 84 | 15 May 2021 | 14 |
|  | Gqeberha (PE) | 15 August 2020 | 14 | 12 October 2020 | 11 | 12 October 2020 | 11 |
| Tree season | Cape Town* | 2 August 2020 | 23 | 4 September | 326 | 11 May 2021 | 47 |
|  | Johannesburg | 20 July 2020 | 22 | 1 September 2020 | 397 | 24 March 2021 | 16 |
|  | Pretoria | 12 August 2020 | 25 | 4 September 2020 | 154 | 24 October 2020 | 53 |
|  | Durban | 3 August 2020 | 34 | 4 February 2021 | 43 | 21 February 2021 | 15 |
|  | Bloemfontein | 6 August 2020 | 37 | 9 September 2020 | 940 | 21 October 2020 | 19 |
|  | Kimberley | 21 August 2020 | 27 | 24 December 2020 | 61 | 28 December 2020 | 27 |
|  | Gqeberha (PE) | No counts >15 |  | No counts >15 |  | No counts >15 |  |

*The Cape Town spore trap power supply was faulty for a period during 2020/2021, resulting in missing data during the grass season and the peak tree season. Pollen measurements from 2010-2018 were thus used to calculate the Cape Town grass season start, peak, and end dates, as well as the peak date for trees.

**Appendix Figure 1:** The weed pollen types contributing more than 3% to the Annual Pollen Index (API) in A) South Africa, B) Cape Town, C) Johannesburg, and D) Pretoria between 2019-2021.

**Appendix Figure 2:** The weed pollen types contributing more than 3% to the Annual Pollen Index (API) in A) Bloemfontein, B) Kimberley, C) Durban, and D) Gqeberha between 2019-2021.

**Appendix Figure 3:** The percentage contribution of each sampling site to the Annual Pollen Index of trees, weeds, and grass pollen in South Africa (2019-2021). The sites are Cape Town (CPT), Johannesburg (JHB), Pretoria (PTA), Bloemfontein (BFN), Kimberley (KMB), Durban (DBN), and Gqeberha (PE).
