# Supplementary material for: The South African Pollen Monitoring Network: Insights from 2 years of national aerospora sampling (2019–2021)
Source: Clin Transl Allergy. 2023 Nov 1;13(11):e12304. doi: 10.1002/clt2.12304 (PMC10620116; doi:10.1002/clt2.12304)
Supplement: Supplementary file 2 — Supporting Information S2 [file CLT2-13-e12304-s002.docx]

## Supplementary Material

## Site descriptions

**Cape Town**

Cape Town (CPT) suburbs are scattered across the narrow Cape Peninsula and extend up the western and eastern coastlines. The topography is complex as Table Mountain rises steeply to a height of 1 086 metres from its position close to the port of Cape Town in Table Bay and there are two coastlines around the Cape Peninsula. These features give rise to different climatic conditions within a small area. The climate is Mediterranean with winter rainfall and warm, dry summers but rainfall varies from 350 mm at the western coast to > 2 000 mm per year on the highest mountain summits (1,2). The Cape Town spore trap is situated on a rooftop (ca. 8 m above ground) at the South African Astronomical Observatory (SAAO) in the mid-Peninsula area. The City of Cape Town is situated within a global biodiversity hotspot in the south-western corner of the species-rich Cape Floristic Region. Indigenous vegetation types in the surroundings of Cape Town include Peninsula Shale Renosterveld, Peninsula Sandstone Fynbos, and Southern Afro-temperate Forest on the slopes of Table Mountain (2). Typical indigenous fynbos taxa include Ericaceae, Restionaceae, Proteaceae, and other ericoid shrubs (1). Elytropappus (renosterbos), Oxalis, Stoebe, Ericaceae, diverse grasses as well as few shrubs like Searsia and Myrsine are typical elements of renosterveld (1). However, the natural vegetation is degraded and reduced due to urban development, with neophytes like Platanus, Pinus, Quercus, and Australian Acacia being common (2,3). The vegetation immediately surrounding the SAAO includes an indigenous garden with fynbos species, but exotic trees like Eucalyptus, Schinus and cypress trees, as well as indigenous trees like Searsia and Podocarpus, are also present (3).

**Johannesburg**

Johannesburg (JHB) is South Africa’s most populous city with 8 million inhabitants living in the greater Johannesburg area. It is regarded as one of the most extensive man-made urban forests globally (4,5). The regional climate is warm-temperate subtropical and has a typical summer rainfall pattern with warm, wet summers and dry, cool winters. Johannesburg is located within the Grassland biome characterized by grasses (Poaceae). The dominant grass species is *Themeda triandra* and diverse herbs and shrublets include species of Asteraceae (among others, *Stoebe vulgaris* (4)), Rubiaceae, Acanthaceae, and Euphorbiaceae (1). The vegetation northeast of the spore trap location consists of various trees, including *Celtis*, *Englerophytum*, *Canthium*, *Vangueria*, *Searsia*, *Senegalia/Vachellia* and the genus *Buddleja*. Exotic trees in the area include Australian taxa like *Eucalyptus*, *Callistemon, Acacia* and *Jacaranda mimosifolia* from South America. Many Eurasian and North American taxa, e.g., P*latanus*, *Morus*, *Liquidambar*, *Betula*, *Carya illinoiensis*, *Fraxinus* and *Quercus* are also present (6,7,5). The Johannesburg spore trap is located on the roof of the Richard Ward Building (ca. 12 m above ground), School of Chemical and Metallurgical Engineering, Braamfontein Campus East, University of the Witwatersrand. The surrounding vegetation consists of ornamental garden plants on the densely built campus, with some indigenous plants like *Podocarpus*, *Aloe*, *Clivia*, *Strelitzia*, *Combretum* and *Bauhinia* present.

**Pretoria**

The Pretoria (PTA) spore trap is located on the roof of the Energy Centre building on the campus of the Council for Scientific and Industrial Research (CSIR) in the Gauteng province. The site is an urban background site and the CSIR campus is near the N1 highway situated in a residential area of the city. The Köppen-Geiger climate classification for the City of Tshwane is transitional between BSh (arid, steppe, hot) and Cwb (temperate, dry winter, warm summer) (8). The surrounding vegetation on the CSIR campus is a variety of mostly indigenous trees and grasses. The baseline vegetation for the site is Marikana Thornveld (1) and many of the common species typical of this vegetation are found on the CSIR campus. This includes tree species such as, *Senegalia burkei, Vachellia karroo, V. nilotica, Combretum molle, Searsia lancea, Ziziphus mucronata, Celtis africana*, and grass species such as, *Elionurus muticus, Eragrostis lehmanniana, Setaria sphacelata,* and *Themeda triandra* (1). Industrial development has transformed the landscape and alien invasive plants like Australian *Acacia* occur in high densities. Other exotic plants in the area include *Pinus*, *Quercus*, *Platanus,* and *Morus* (6). Pretoria is also known as Jacaranda City, as 70 000 exotic *Jacaranda* tree species were planted throughout the urban landscape.

**Bloemfontein**

Bloemfontein (BFN) is in the central parts of South Africa in the southern Highveld, bordering on the semi-arid region of the Karoo. This city lies within the Grassland biome (1), divided into different vegetation types. The dominant vegetation types in the vicinity are Bloemfontein Dry Grassland (Gh 5), Central Free State Grassland (Gh 6), Winburg Grassy Shrubland (Gh 7), Bloemfontein Karroid Shrubland (Gh 8) and Highveld Alluvial Vegetation (Aza 5) along the nearby Modder River. The area is generally flat with occasional hills (koppies in Afrikaans), or incised river valleys and the general vegetation is Dry Highveld Grassland. The vegetation is mostly controlled by the annual rainfall that forms an east to west gradient of decreasing moisture content across the Highveld. Although the vegetation is mostly dominated by grasses there is distinctive shrubland that occurs on rocky slopes and outcrops where the soils are shallow and stony. Bloemfontein experiences a semi-arid climate (Köppen: BSk), with hot summer days and cooler, dry winters – often with frosts. Rainfall is restricted to the summer months and is of convectional origin, peaking in late summer. The Bloemfontein spore trap is located on the roof of the Biology Building (ca. 11 m above the ground) of the University of the Free State’s Bloemfontein campus. The campus gardens are characterised by indigenous plants species, e.g., Ziziphus mucrontata, Euclea crispa, Olea europaea subsp. africana, Searsia burchelli, S. erosa, S. lancea, S. penduline, Lycium cinereum, Buddleja saligna, Combretum erythrophyllum, Podocarpus henkelii, Vachellia karroo, V. tortilis, Themeda triandra, Eragrostis obtusa and E. superba, as well as exotic species such as Betula pendula, Eucalyptus globulus, Ginko biloba, Platanus acerifolia, Quercus ruber and various species of cypress and pine.

**Kimberley**

Kimberley (KMB) is a diamond mining town at the north-eastern border of the Northern Cape province. It is located 176 km from Bloemfontein in the Grassland and Savanna biome. Kimberley is in a summer rainfall region, with a short and very cold dry winter (-8 °C) and a long hot summer with frequent thunderstorms. The average max temperature is 26.1 °C, however, it is not uncommon to experience temperatures up to 39 °C and higher during summer months. Kimberley is set in a relatively flat landscape with no prominent topographic features. *Themeda triandra* is the most dominant grass species found in the Grassland biome. However, prominent species like *Eragrostis curvula, Cymbopogon pospischilli, Setaria sphacelata, Digitaria eriantha, Hyparrhenia hirta* and *Cynodon dactylon* also occur throughout the region (9). The Kimberley spore trap is located on the roof of Mediclinic Gariep Hospital (12.1 m above ground) at the southern outskirts of town. The hospital is bordered from the southern and western sides by natural veldt. The surrounding suburban gardens lie to the north and eastern side and contain indigenous plant species such as multiple thorn trees (*Vachellia/* *Senegalia*), blink-blaar-wag-'n-bietjie (*Ziziphus* *mucronata*), camphor bush (*Tarchonanthus*), karee (*Searsia*), hackberry (*Celtis*), bushwillow (Combretaceae) and olive (Oleaceae). Exotic trees include weeping willow (*Salix*), Australian pine (*Casuarina*), mulberry (*Morus*), poplar (*Populus*) and plane (*Platanus*).

**Durban**

Durban is characterized by a subtropical climate, with most rain falling in the summer months. Diverse geological, topographical, and climatic conditions have led to a myriad of terrestrial and aquatic ecosystems (10), with Durban forming part of the Maputaland-Pondoland-Albany global biodiversity hotspot (11). Many of the surrounding gardens include a diversity of species both indigenous and endemic to southern Africa, but not necessarily to Durban. This, plus the lore of exotic ornamental species, includes frangipani (*Plumeria spp.*), African Flame Tree (*Spathodea campanulata*), Flamboyant (*Delonix regia*), and *Tibouchina granulosa* (12), results in a mosaic of indigenous, invasive, and alien plant species. The more common tree species in the gardens are palms (*Hyphaene coriacea*, *Raphia australis,* and *Phoenix reclinata*), tree fern (*Cyathea dregei*), and tree species such as cabbage tree (*Cussonia*), coral trees (*Erythrina latissima* and *Erythrina lysistemon*), forest fever berry (*Croton sylvaticus*), Natal Mahogany (*Trichilia emetica*), flat-crowned albizia (*Albizia adianthifolia*) and several figs (*Ficus*). Commonly planted fruit trees in the area include pawpaw (*Carica papaya*), banana (*Musa x paradisiaca*), avocado (*Persia gratissima*), and mango (*Manifera indicae*) (12). Common mulberry (*Morus alba*) and guava (*Psidium guajava*) have become naturalised and are problematic invasive species (12). It must also be mentioned that Durban, due to its humid subtropical climate, is the site with the highest fungal spore loads in South Africa – as recorded by the South African Pollen Monitoring Network. The Durban spore trap is installed on the roof of the Westville Hospital (ca. 12-15 m above ground).

**Gqeberha (PE)**

Gqeberha, formally known as Port Elizabeth (PE), is a major seaport city located along the eastern coastline of South Africa within the Eastern Cape province. The proximity to the ocean has a moderating effect on the climate with cool winters and warm summers. Wind speeds can often be gale force and wind directions vary but are most often from the west and east during summer. The Gqeberha spore trap was installed on the rooftop of Nelson Mandela University’s Ocean Sciences department building (ca. 12 m above ground). This building is located on the edge of Nelson Mandela University’s Nature Reserve. Therefore, the spore trap samples the immediate urban flora and the natural vegetation found within the adjacent reserve. The vegetation of the coastal city of Gqeberha reflects the mosaic nature of the region, being a confluence point of five of the nine biomes of South Africa (Main text Figure 1). Taylor and Morris (13) recognized five major vegetation categories within the region. These are: (1) *Scaevola thunbergii* pioneer vegetation of littoral dunes, and *Ficinia lateralis* Sedgeland of calcrete gravel, (2) *Olea exasperata* Bush, *Pterocelastrus tricuspidatus* Bushclumps and Dune woodland, (3) Sundays River Scrub, (4) Fynbos of calcrete areas and (5) *Themeda triandra* Grassland. Common indigenous plant species found within the immediate vicinity of the Gqeberha spore trap include various *Aloe* species, spekboom (*Portulacaria afra*) coral trees (*Erythrina latissima* and *Erythrina lysistemon*), and coastal fynbos shrubs such as blombos (*Metalasia muricata*). Exotic trees found near the trap include palms, *Pinus,* and gums (*Eucalyptus*). The invasion of the area by *Acacia cyclops* (Australian Wattle) is also widely seen.

## Method limitations

Throughout the study, pollen concentrations in the city of Gqeberha remained extremely low – possibly resulting from frequent strong wind currents and onshore wind. A new sampling site at a more optimal location within this city should be investigated in the future. The limited seasonality, low diversity and low pollen concentrations found in Gqeberha throughout the year could likewise be attributed to rainfall, as this site is characterised by aseasonal rains. In the current Cape Town dataset, spore trap malfunction resulted in gaps in the data during the grass flowering season of 2020 and 2021 which might also affect the comparison with historical data. Besides specific local factors like trap location, topography, and climate, there are also general limitations to pollen monitoring, e.g., pollen identification through light microscopy. Limitations to this method include difficulty distinguishing between the pollen from closely related taxa. For example, the pollen ornamentation is remarkably similar throughout the Poaceae (14), and therefore Poaceae genera and species cannot be distinguished based on pollen morphology alone. Instead, molecular analysis of the pollen grains can be done to accurately identify the grass pollen present at different sampling locations (15,16), and in doing so, we can isolate the specific seasons and flowering times for each South African grass species. This is relevant information for the effective treatment of patients who are sensitised to different grass species. In addition, Scanning Electron Analysis (SEM) studies examining the pollen sculpture of comparative pollen material are an option that can help to identify closely related plant taxa - also improving future pollen identification under the light microscope.

This pilot study focussed on the most populous cities spread out across the country, however, a large proportion of people in South Africa live in rural environments and future work of the SAPNET project will aim to sample in these locations. Sites should thus reflect a diversity of climates, topography, vegetation types and differences in land-use (e.g., urban gardens, parks and agricultural land) to capture potential differences between geographic regions. The differences observed in closely located sites also illustrate the need to extend the network to other previously unsampled South African cities and towns, to obtain representative counts for all areas.

**Summary Pollen Calendars for South Africa**

**Supplementary Figure 1:** Pollen Calendars showing the season of A) Trees, B) Weeds, and C) Grass across the whole of South Africa. The average weekly pollen concentrations (2019-2021) are displayed as coded levels: 0 = 0-3; 1 = 3-10; 2 = 10-30; 3 = 30-100; 4 = >100 pollen grains/m^3^ (adapted from 17).

## References

1. Mucina L, Rutherford MC (eds). The Vegetation of South Africa, Lesotho and Swaziland. Strelitzia 19. Pretoria: South African Biodiversity Institute; reprint 2011.

2. Rebelo AG, Holmes PM, Dorse C, Wood J. Impacts of urbanization in a biodiversity hotspot: Conservation challenges in Metropolitan Cape Town. *S Afr J Bot* 2011;77:20-35.

3. Berman D. Variations in pollen and fungal spore air spora: an analysis of 30 years of monitoring for the clinical assessment of patients in the Western Cape. Cape Town: University of Cape Town; 2018.

4. Schäffler A, Swilling M. Valuing green infrastructure in an urban environment under pressure - The Johannesburg case. *Ecol Econ* 2013; 86:246-257.

5. Symes CT, Roller K, Howes C, Lockwood G, van Rensburg BJ. Grassland to Urban Forest in 150 Years: Avifaunal Response in an African Metropolis. In: Murgui E, Hedblom, M (eds). Ecology and Conservation of Birds in Urban Environments. Cham: Springer International Publishing; 2017.

6. Glen H, van Wyk B. Guide to Trees Introduced into Southern Africa. Cape Town: Struik Nature; 2016.

7. Turton A, Schultz C, Buckle H, Kgomongoe M, Malungani T, Drackner M. Gold, Scorched Earth and Water: The Hydropolitics of Johannesburg. *Int J Water Resour D*, 2006;22:313-335.

8. Engelbrecht CJ, Engelbrecht FA. Shifts in Köppen-Geiger climate zones over southern Africa in relation to key global temperature goals. *Theor Appl Climatol* 2016;123:247-261.

9. O`Connor, TG, Bredenkamp, GJ. Grassland. In: Cowling RM, Richardson DM, Pierce SM (eds). Vegetation of Southern Africa. Cambridge University Press; 1997.

10. Govender N. Durban Climate Change Strategy Introductory Report Theme Biodiversity. Environmental Planning and Climate Protection Department, Durban; 2013.

11. Steenkamp Y, van Wyk AE, Victor JE, Hoare DB, Dold AP, Cowling RM, Smith GF. Maputaland-Pondoland-Albany. In: Mittermeier RA, Hoffmann M, Pilgrim, JD, Brooks TB, Mittermeier CG, Lamoreux JL, da Fonseca G (eds). Hotspots revisited: Earth’s biologically richest and most endangered ecoregions. Mexico City: Cemex; 2004

12. Cadman A, Dames JF. Airspora of Durban: a sub-tropical, coastal South African city. *Grana* 1993;32(6): 372-375.

13. Taylor HC, Morris JW. A brief account of coastal vegetation near Port Elizabeth. *Bothalia* 1981;13(3/4):519-525.

14. Gibbs Russell GE, Watson L, Koekemoer M, Smook L, Barker NP, Anderson HM, Dallwitz MJ. Grasses of Southern Africa. Memoirs of the Botanical Survey of South Africa. Pretoria: National Botanical Gardens; 1990.

15. Ghitarrini S, Pierboni E, Rondini C, Tedeschini E, Tovo GR, Frenguelli G, et al. New biomolecular tools for aerobiological monitoring: Identification of major allergenic Poaceae species through fast real-time PCR. *Ecol Evol.* 2018;8(8):3996-4010.

16. Leontidou K, Vernesi C, Vokou D, De Groeve J, Cristofolini F, Cristofori A, et al. Efficient and sensitive identification and quantification of airborne pollen using next-generation DNA sequencing. *Sci Rep.* 2019;15(1):8-16.

17. Potter C, Cadman A. Pollen allergy in South Africa. *Clin Exp Allergy* 1996; 26:1347-1354.
